# The Treatment of Acute Intestinal Obstruction

**Published:** 1898-11-12

**Authors:** 


### THE TREATMENT OF ACUTE INTESTINAL OBSTRUCTION.

In the course of an address on "Some Rudiments of Intestinal Surgery," delivered before the Midland Medical Society, Mr. Treves made some important remarks upon certain essentials in the treatment of acute intestinal obstruction. In such cases the occlusion of the bowel is by no means either the most serious or the most essential element in the progress of events. In a really acute case death will take place in seven days. Yet the obstruction of the bowel *per se* is not an acutely serious thing, for the intestine may be blocked for a week, or for two weeks, or even for three weeks without any very distressing or alarming symptoms occurring. Again, the clinical manifestations in a case of acute intestinal obstruction are due to conditions other than those arising from obstruction of the lumen of the gut; and, finally, there is a period in the progress of such cases at which the relief of the obstruction will not save the patient's life.

In the course of a case of acute intestinal obstruction, it is possible to recognise three stages. In the first stage —that of the onset of the trouble—the symptoms are those of intense abdominal pain, collapse, and vomiting. Take the case of the snaring of a loop of ileum by a peritoneal band. The symptoms are in no way due to the fact that the bowel has become abruptly blocked. They depend upon the circumstance that a sudden and intense impression has been made upon exquisitely sensitive abdominal nerves. They are, indeed, purely nervous, and, as it is very important to observe, they have little or no diagnostic character, but are common to a series of intra-abdominal accidents, and may equally well mark the passage of a gall-stone, the torsion of an ovarian tumour, the perforation of an ulcer of the stomach, or the rupture of a pericæcal abscess. In what may be termed the second stage in a case of acute obstruction, the symptoms attain more individuality, and depend without doubt mainly upon the mechanical obstacle in the bowel. The pain is to a large extent due to disordered and futile peristaltic movement, the absolute constipation depends upon the blocking of the intestine, and the persistent vomiting is in the main due to the same cause; while the distension of the abdomen is largely due to accumulation—to mere inability of the contents to escape. But in the third or final stage of acute intestinal obstruction the symptoms again have but little to do with the obstruction as such. They are symptoms of poisoning, and the patient who dies unrelieved dies poisoned, the septic matter which kills him being derived from his own intestine. It is this rapid onset of septicæmia which makes the operative treatment of acute intestinal obstruction so futile in the majority of cases, for death may occur even when the obstruction has been readily discovered and removed.

Those who wish to advance the therapeutics of intestinal obstruction must first of all grapple with this terrible element of intestinal septicæmia. Doubtless in many of these cases there is, before the end comes, an infection of the peritoneum; but the infection starts from the bowel, and in the bowel the process of antisepsis must commence. From a practical point of view, then, the treatment of acute intestinal obstruction appears to demand two things of the surgeon; first, an early operation; and second, the evacuation of the bowel after the strangulation has been relieved.

Any operation, then, that is undertaken must not be considered complete until the bowel has been emptied, which may most conveniently be done by bringing the most distended coil of intestine to the parietal incision, and evacuating it by means of a small glass tube, which is inserted into the gut and secured to the bowel wall by a single encircling thread. The small resulting artificial anus thus produced may be closed by a subsequent operation, carried out some weeks after the original proceeding. Mr. Treves says that this measure has had the effect, in his own practice, of reducing the mortality of this operation to one-half of its original rate. In fact, records of cases of acute obstruction treated by operation show that by far the best results have occurred when the surgeon has simply made a small abdominal incision and has drawn out the first distended coil which has presented itself, and has been content to open it and fix it in position—the cause of the obstruction being ignored and not even sought for. In any case, no operation for really acute intestinal obstruction is to be looked upon as complete until the bowel has been evacuated of its contents. And this is the great lesson.

